# Peripheral blood microRNAs expression is associated with infant respiratory syncytial virus infection

**DOI:** 10.18632/oncotarget.19364

**Published:** 2017-07-18

**Authors:** Shouyi Wang, Pin Liu, Pu Yang, Junwen Zheng, Dongchi Zhao

**Affiliations:** ^1^ Department of Pediatrics, Children’s Digital Health and Data Center, Zhongnan Hospital of Wuhan University, Wuhan, China

**Keywords:** respiratory syncytial virus, peripheral blood, miRNA, infants, inflammatory response, Immunology and Microbiology Section, Immune response, Immunity

## Abstract

MicroRNAs respond to the inflammatory responses induced by RNA virus infection. In this study, we investigated the specific microRNA profile in the peripheral blood of infants infected with respiratory syncytial virus (RSV). Blood specimens were analyzed using microRNA microarrays, followed by quantitative RT-PCR. A specific microRNA profile in the peripheral blood of RSV-infected infants was identified for the first time. MiR-106b-5p, miR-20b-5p, and miR-342-3p were upregulated, while miR-320e, miR-320d, miR-877-5p, miR-122-5p, and miR-92b-5p were downregulated. Pathway analysis indicated that the dysregulated microRNAs were involved in inflammatory and immune responses, including Wnt, TGF-β, insulin, and T and B cell receptor signaling. These results demonstrate that RSV infection associates with a distinct microRNA fingerprint and suggest that RSV induces inflammatory responses in infants.

## INTRODUCTION

Respiratory syncytial virus (RSV) is an enveloped negative strand RNA virus, which belongs to the *Paramyxoviridae* family. Infants with RSV infection often develop severe bronchiolitis or pneumonia [1]. The lack of understanding of the host-virus interface has hindered prevention and treatment methods, as well as a successful RSV vaccine development [2]. Even though recent antiviral efforts have begun to inhibit virus replication by targeting host pathways and using interfering RNAs [3-6], regulation of the host immune responses resulting in bronchiolitis remains a challenge.

Mature microRNAs (miRNA) are non-coding transcripts 18 to 25 nucleotides in length, which modulate protein expression at the post-transcriptional level [7]. The miRNA gene family makes up a global regulatory network controlling homeostasis, cell proliferation, differentiation, cell migration, disease progression, and inflammatory responses [8-10]. Many viruses can make use of the host processing machinery for their biogenesis by encoding miRNAs [11]. Viral infections can also influence host miRNA production. In addition, host miRNAs can regulate the viral life cycle, and target the viral messenger RNA [12].

Recent studies have indicated that miRNAs regulate the inflammatory response associated with RNA virus infection. Let-7c, upregulated in influenza virus-infected epithelium, may inhibit influenza virus replication by directly targeting the viral gene product [13]. RSV non-structural protein-1 modifies mir-24 expression via transforming growth factor beta (TGF-β) [14]. The lack of downregulation of miR-125a and miR-429 in severe RSV infection may be associated with RSV manifestations [15]. MicroRNA-221 modulates RSV replication in human bronchial epithelium by targeting nerve growth factor expression [16].

Understanding the changes in miRNA expression profiles and identifying the targets genes and their contribution to viral infection may help elucidate novel mechanisms of host-virus interaction [17-18]. In this study, we analyzed the miRNAs fingerprint in whole blood of infants with RSV.

## RESULTS

### Clinical characteristics

Five males and five females were included in the study. The sex ratio was similar in the two sets of samples. The diagnosis of RSV infection was based on clinical manifestation, physical signs of lung, laboratory tests, and examination (Table 2). All patient samples were acquired within 7 days of infection onset.

**Table 1:** Computational algorithms and methods for miRNA target prediction

Name	Web link
miRanda	http://www.microrna.org
TargetScan	http://genes.mit.edu/targetscan
PicTar	http://pictar.mdc-berlin.de
DIANA-microT	http://diana.pcbi.upenn.edu/cgi-bin/micro_t.cgi
RNAhybrid	http://bibiserv.techfak.uni-bielefeld.de/rnahybrid
RNA22	http://cbcsrv.watson.ibm.com/rna22.html
PITA	http://genie.weizmann.ac.il/pubs/mir07/mir07_data.html
miRDB	http://mirdb.org
miRWalk	http://www.ma.uni-heidelberg.de/apps/zmf/mirwalk/
KEGG GOmir	http://www.genome.jp/kegg/ http://www.bioacademy.gr/bioinformatics/projects/GOmir

**Table 2:** Clinical characteristics of patients with RSV and healthy controls

	RSV1	RSV2	RSV3	RSV4	RSV5	CTL1	CTL2	CTL3	CTL4	CTL5
Gender	male	male	female	female	male	female	male	female	female	male
Age (months)	2.5	3.5	3.5	3	7	12	1	1	6.5	3
Onset time (days)	3	5	6	4	1	/	/	/	/	/
Pulmonary auscultation	wheeze	wheeze	wheeze	wheeze	wheeze	clear	clear	clear	clear	clear
Respiratory rate (bpm)	38	35	32	34	30	26	35	33	31	30
Oropharyngeal swab	+	+	+	+	+	-	-	-	-	-
Chest X-ray	/	bronchitis	bronchitis	bronchitis	bronchitis	/	/	/	/	/
White blood count(10^9^/L)	9.79	8.61	7.2	8.81	6.46	9.28	10.48	8.93	5.75	8.35
Neutrophil granulocyte%	33.1	22.8	15.4	31.3	25	58.1	34.3	24.2	57.5	35.3
Lymphocyte%	49.1	63.4	75.7	60.5	65.4	36.5	54.7	56.7	33.2	50.9

### miRNA expression profiles

**Figure 1:**
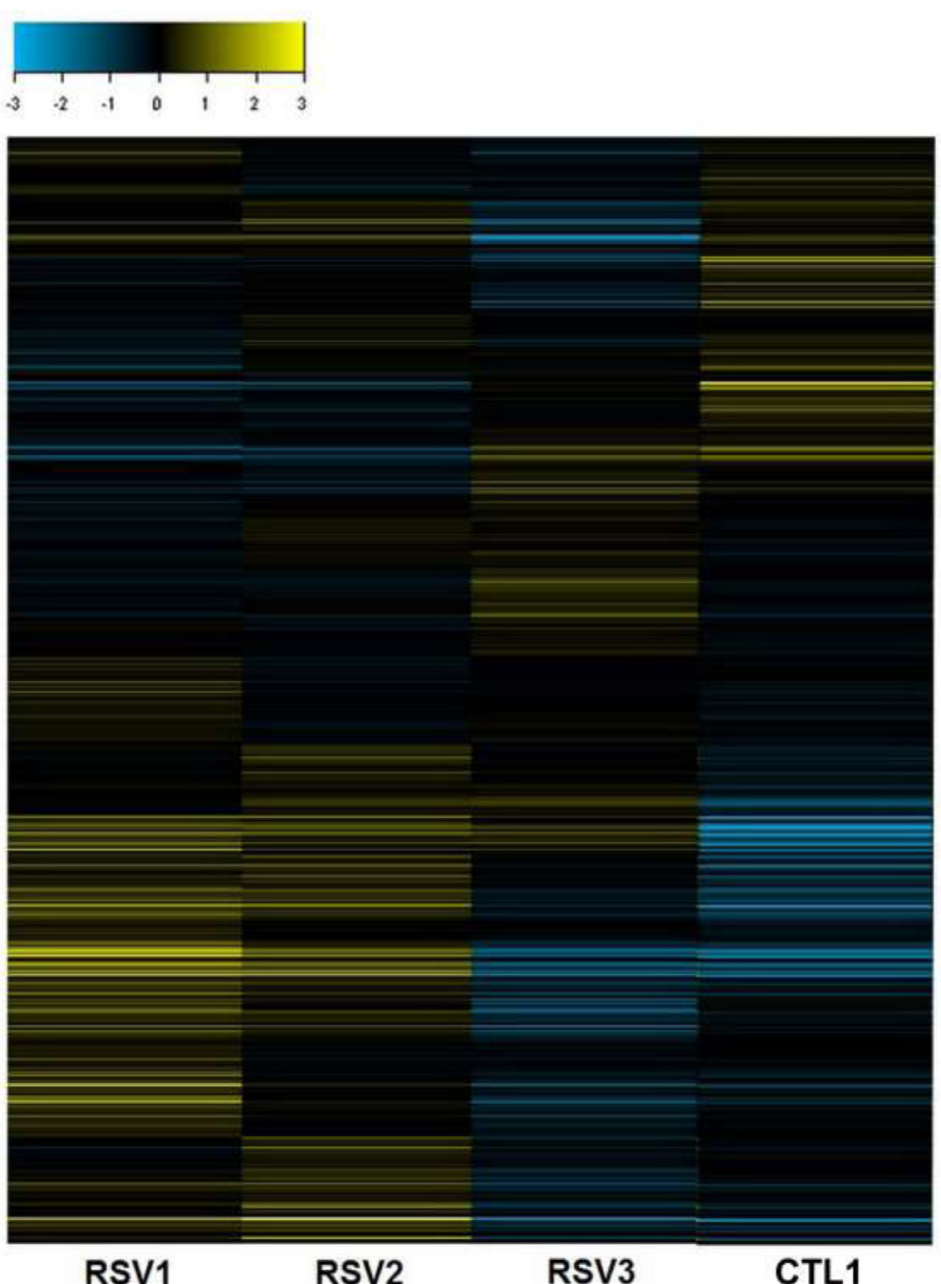
RSV regulated microRNAs expression in peripheral blood This heat figure was generated using comparison analysis based on signal value ratio method; it provides an overall picture of the impact of RSV infection on the peripheral blood miRNAs as compared to healthy control blood. Data were partitioned between RSV and control (CTL1) samples during the hierarchical clustering and organized into blocks of columns. The rows represent probe sets in each of the arrays, and within each row, the blue shaded areas indicate lower expression of the specific miRNA, whereas the yellow shaded areas indicate higher expression. Black and darkly shaded areas indicate similar expression between infected and non-infected samples. The Euclidean clustering method was used for array data analysis. The expression index is shown in the left upper corner.

Differential expression analysis of miRNA microarrays between RSV patients and healthy controls indicated that 37 miRNAs were upregulated-fold-change ≥ 2, while 24 miRNAs were downregulated (fold-change ≤ 0.5), and 72 miRNAs (fold-change=0.5 ∼ 2.0) were not changed. The miRNA microarrays analysis data are shown in a hierarchical clustering (Figure 1). From the differentially expressed miRNAs, the top five upregulated miRNAs and six downregulated miRNAs were selected for further validation (Table 3).

**Table 3:** Differential expression of microRNAs between RSV and healthy control groups

MicroRNAs	RSV VS CTL (fold-change)
hsa-miR-106b-5p	85.03747
hsa-miR-20b-5p	67.83043
hsa-miR-181a-5p	31.82493
hsa-miR-652-3p	12.15890
hsa-miR-342-3p	11.55260
hsa-miR-320e	0.12787
hsa-miR-320d	0.24643
hsa-miR-877-5p	0.27453
hsa-miR-122-5p	0.31253
hsa-miR-92b-5p	0.35467
hsa-let-7c-5p	0.36293

RSV=Respiratory syncytial virus infection sample No.1, 2, 3; CTL=Control sample. The fold-change of RSV =average fold-change of (RSV1, RSV2, RSV3). Fold-change≥2.0 indicated upregulation, fold-change≤0.5 indicated downregulation.

### qRT-PCR validation of selected miRNAs

**Figure 2:**
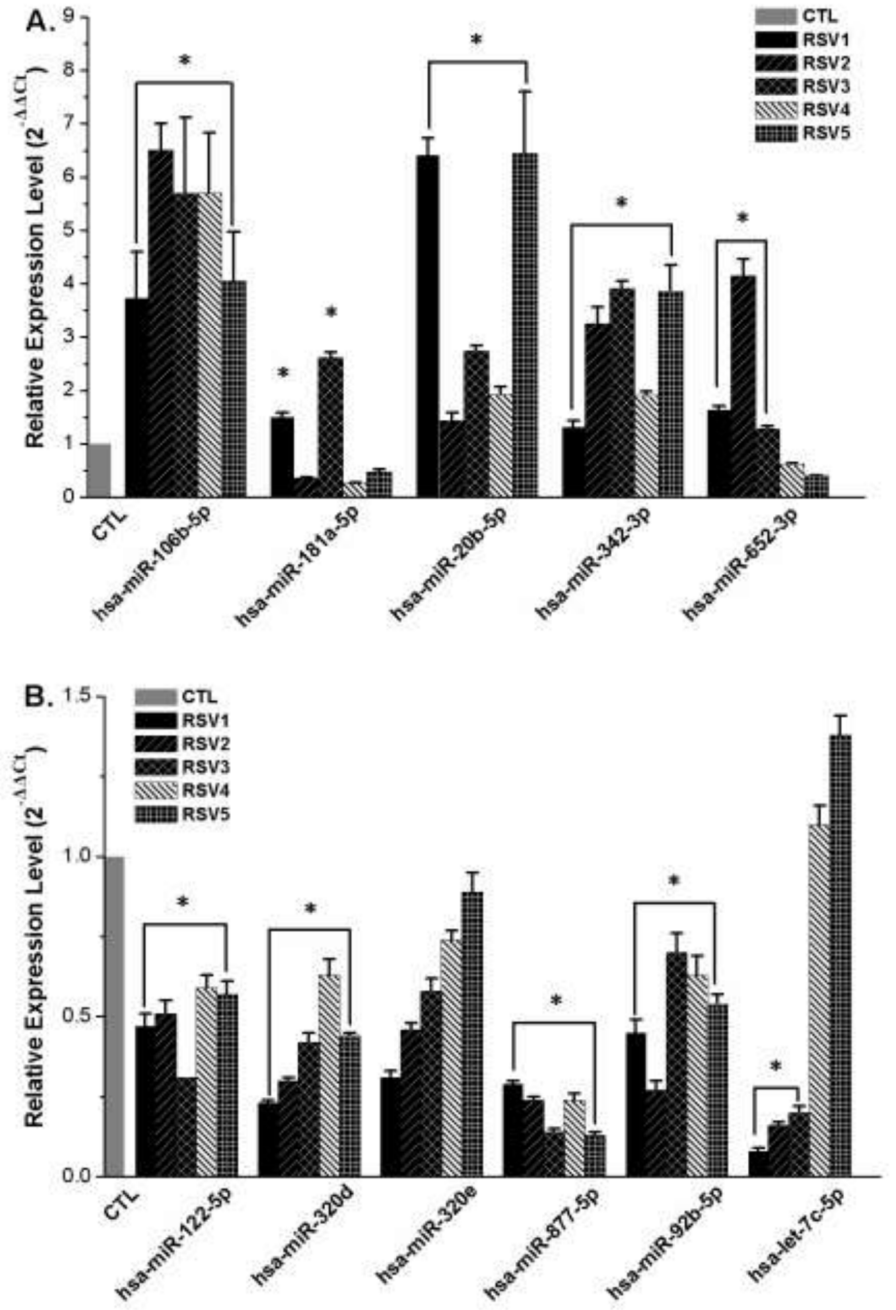
Validation of miRNA microarray findings by quantitative qRT-PCR Grey column represents miRNAs relative expression level of control samples (2^-ΔΔCt^=1.0). Other color columns represent the selected miRNAs relative levels of each RSV infection sample. **A.** Relative expression of five upregulated miRNAs (miR-106b-5p, miR-181a-5p, miR-20b-5p, miR-342-3p and miR-652-3p) in microarray. **B.** Relative expression of six downregulated miRNAs (miR-122-5p, miR-320e, miR-320d, miR-877-5p, miR-92b-5p and let-7c-5p) in microarray. Asterisk indicates the significant differences between RSV infection and control groups (*P* < 0.05).

The validation qRT-PCR results are shown in Figures 2A and 2B. Expression of three of the five upregulated miRNAs was significantly different between the RSV group and the control group. However, expression of miR-181a-5p in RSV2, RSV4, RSV5, and miR-652-3p in RSV4 and RSV5 was not consistent with the microarray data. Expression of four of the six downregulated miRNAs (miR-122-5p, miR-320d, miR-877-5p and miR-92b-5p) showed a similar trend as that of the microarrays. However, there was no significant change of miR-320e in all five RSV infected cases, and let-7c-5p was downregulated in RSV1, RSV2, and RSV3, while there was no difference in RSV4 and RSV5. These results demonstrated that most qRT-PCR results (75%) of miRNA expression were consistent with microarray analyses.

### Prediction of target miRNAs function

By using ten different algorithms DIANAmT, miRanda, miRDB, miRWalk, RNAhybrid, PICTAR4, PICTAR5, PITA, RNA22, and Targetscan, we obtained a list of genes predicted to be targeted by miR-106b-5p, miR-20b-5p, miR-342-3p, miR-122-5p, miR-320d, miR-877-5p, and miR-92b-5p. If one predicted gene was validated by one database/algorithm, it marked one point. The higher the score the gene received, the more reliable it was. Gene ontology (GO) enriched function analysis and KEGG pathway analysis indicated that miR-106b-5p, miR-20b-5p, miR-342-3p, miR-877-5p, miR-122-5p, miR-320d and miR-92b-5p were involved in many common pathways (Table 4). A large number of pathways were associated with inflammatory and immune processes, such as insulin signaling, TGF-beta signaling, Wnt signaling, T and B cell receptor signaling, and Fc epsilon RI signaling pathway. Natural killer cell mediated cytotoxicity was the common pathway of miR-106b-5p, miR-20b-5p, and miR-342-3p.

**Table 4:** Common pathways of seven validated differential expression microRNAs by KEGG analysis

Pathway term	Count	*P* value
Focal adhesion	35	6.42E-17
TGF-beta signaling pathway	22	7.64E-15
T cell receptor signaling pathway	21	9.17E-12
MAPK signaling pathway	33	1.33E-11
ErbB signaling pathway	17	6.83E-10
Wnt signaling pathway	21	5.43E-09
Leukocyte transendothelial migration	17	1.18E-07
Insulin signaling pathway	18	1.61E-07
Cytokine-cytokine receptor interaction	25	3.62E-07
Jak-STAT signaling pathway	14	2.30E-04
Toll-like receptor signaling pathway	10	9.27E-04
B cell receptor signaling pathway	8	1.72E-03
Fc epsilon RI signaling pathway	7	9.09E-03

Note: Seven validated differential expression microRNAs were miR-106b-5p, miR-20b-5p, miR-342-3p, miR-877-5p, miR-122-5p, miR-320d and miR-92b-5p. Count: Number of potential target genes in the pathway; *P* value is significant at the 0.05 level.

**Figure 3:**
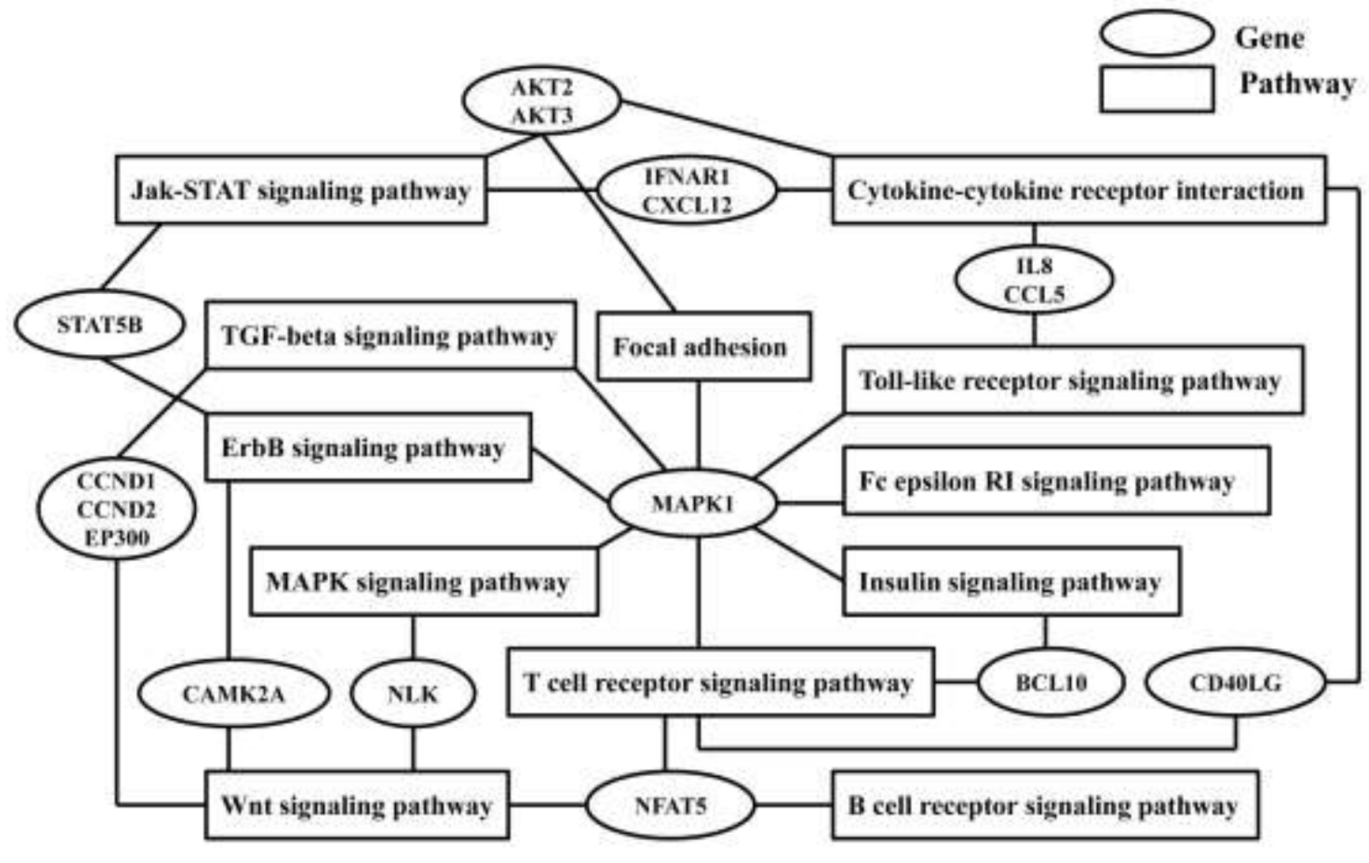
Common genes and relevant pathways’ crossing network of seven validated differential expression microRNAs

The common pathways’ crosstalk is displayed in Figure 3. There were twelve pathways and sixteen predicted genes in the crosstalk network. MAPK1 was involved in eight pathways, and NFAT5 participated in regulating three pathways. These signaling pathways are involved in inflammation and immune responses, including TGF-beta signaling, Wnt pathway, insulin signaling, Toll-like receptor signaling, T and B cell receptor signaling, and Fc epsilon RI signaling pathway.

## DISCUSSION

In this study, miRNA expression profiles were analyzed in peripheral blood of infants with acute RSV infection, and differential expression of miR-106b-5p, miR-20b-5p, miR-342-3p, miR-877-5p, miR-122-5p, miR-320e, miR-320d, and miR-92b-5p was confirmed by qRT-PCR. The function analysis revealed that the miRNAs participate in signaling pathways involved in inflammation and immune responses.

Previous study using nasal mucosal samples showed that RSV-positive infants downregulated miR-34b, miR-34c, miR-125b, miR-29c, miR-125a, miR-429 and miR-27b, and upregulated miR-155, miR-31, miR-203a, miR-16 and let-7d [15]. The differential expression of miRNAs in our study may be derived from the different sample site. In addition, clinical samples have a high degree of biological variation, and may differ in the miRNA stability as well.

Viral infections can activate the immune system via the activation of pattern recognition receptors (PRRS). RSV proteins and replication products can be recognized by PRRS, resulting in the activation of a series of transcription factors, including NF-κB, the interferon regulatory factor (IRF)-1, -3 and -7, and the JAK/STAT signaling pathway [24-26]. Through KEGG pathway analysis, we found that the JAK-STAT signaling pathway was a common predicted pathway of eight dysregulated miRNAs. In addition, it was shown that RSV could modulate the host innate immune response by dysregulation of host miRNAs related to the antiviral response, a feature that also affects the memory immune response to RSV [27]. The majority of miRNAs dysregulated in our study was involved in pathways related to the immune and inflammatory responses. Among them, the Wnt signaling pathway, TGF-β signaling, and insulin signaling are involved in inflammatory responses [28], and T cell receptor and Fc epsilon RI signaling are involved in immune responses. Almost half of the dysregulated miRNAs belonged to the miR-17-92 family, which is made of the three paralog miR-17-92, miR-106a-363, and miR-106b-25 clusters. Notably, miR-106b-5p and miR-20b-5p were from the miR-17 seed family [29]. MicroRNA members of the miR-17-92 family were predicted to target Tgfbr2 mRNA upon iNKT cell development. Increased expression of miR-17-92 suppresses TGF-βR II expression and signaling, and activates effector differentiation [30, 31].

Another increased microRNA, miR-342-3p, has been less studied in RSV disease. miR-342-3p is predicted to interact with mRNAs involved in pain signaling, colonic motility, and smooth muscle function [32]. Furthermore, miR-342-3p likely plays a pro-apoptotic role in macrophages through IL-4/STAT6 signaling axis, and provides a negative feedback to IL-4-dependent macrophage proliferation [33]. However, miR-342-5p is upregulated during antiviral response via the IFN-induced transcription factor IRF1, and exerts broad antiviral effects against viruses, such as cytomegalovirus and Influenza A (H1N1) [34].

Interleukin-1 receptor antagonist (IL1RA) is one of the RSV-induced genes [35]. As a target gene of miR-122-5p, interleukin-1 receptor type I (IL1R1) may be activated by increased IL1RA after RSV infection. Toll-like receptor (TLR) 4 is another target gene of miR-122-5p; it is stimulated by the RSV F protein [36]. TLR4-deficient mice infected with RSV exhibit an enhanced disease [37-39]. The acute phase of RSV infection induces expression of IL-13, TGF-β, and IL-6 [40]. The increased production of IL-13 stimulates expression of its receptor IL-13RA1, which further amplifies the Th2 response. Target gene analysis showed that IL-13RA1 is a target gene of miR-877-5p. It was also shown that TGF-β is a major regulator of human neonatal immune responses following RSV infection [22]. TGFB3 and TGFBR1 involved in TGF-β signaling were predicted target genes for miR-877-5p and miR-320d, respectively. Moreover, TGF-β and IL-6 could increase levels of Th17 cells that produce IL-17A, IL-17F, IL-21, and IL-22 [41]. Target gene analysis revealed that several genes associated with Th17 cells: IL-6R, IL-23R, RORγ, STAT3, IL-17A, IL-17RE, IL-17RD, CCR4, and CCR6, which belong to miR-122-5p, miR-320d and miR-877-5p.

In conclusion, we found that three miRNAs were upregulated and five miRNAs were downregulated during RSV infection in infants’ peripheral blood. However, we did not collect blood specimens during recovery phase of RSV infection, which might better reflect the dynamic regulation of host cell miRNAs. Regardless of this limitation, the present data support the hypothesis that miRNAs are the main regulators of the immune response involved in RSV infection.

## MATERIALS AND METHODS

### Subjects

Five RSV infection children were admitted to Zhongnan Hospital of Wuhan University, Wuhan, China, during the RSV season from December 2014 to March 2015. Healthy controls were five healthy children who received regular development examination. Nasopharyngeal aspirates for virus detection were taken from eligible patients on admission, and the specimens were analyzed using a commercial indirect immunofluorescence (IIF) kit (EUROIMMUN, Lübeck, Germany) following the manufacturer’s instructions. Patients with bronchopulmonary dysplasia, chronic lung disease including treated asthma, neurological disease, congenital disorder, hypotonia, failure to thrive, or other specific conditions likely to contribute to a more severe course of disease, were excluded. Whole blood samples were collected within 24 h after a diagnosis of RSV infection. All blood samples were stored at -20^o^C within 4 h following collection prior to analysis for miRNAs.

### RNA extraction and quantitation

Total RNA of peripheral blood (no centrifugation and sedimentation) was extracted by the TRIpure Reagent (BioTeke Corporation, Beijing, China) according to the manufacturer’s instructions. Total RNA purification was performed by mirVana™ miRNA Isolation Kit (AM1561) (Ambion, Austin, TX). RNA quantity and quality were assessed using the NanoDrop^®^ ND-1000 (Thermo Fisher Scientific, Waltham, MA).

### MiRNA microarray hybridization

Three RSV infection samples and one healthy control sample (CTL) were analyzed using miRNA microarrays. The microarray assays were performed by the Affymetrix GeneChip^®^ miRNA 4.0 expression profiling kit at Affymetrix, Inc (Santa Clara, CA). The microarray contained probes for 5214 human miRNAs and generated fluorescent miRNAs with a sample input of 130 ng of total RNA. Labeling was performed using FlashTag™ Biotin RNA Labeling Kit (Genisphere Inc. Hatfield, PA) containing Poly (A) Tailing and FlashTag Ligation. The array hybridization cocktail was prepared. The mixture was incubated at 99 °C for 5 minutes, then 45 °C for 5 minutes. Subsequently, the mixtures were transferred to both septa of a microarray slide, and the arrays were placed into hybridization oven and incubated at 48 °C and 60 rpm for 16 hours. The slides were then washed with washing buffer (Affymetrix Inc, Santa Clara, CA), and scanned immediately by the GeneChip^®^ Scanner 3000 (Affymetrix Inc, Santa Clara, CA). Data collection and quality assessment were performed using Affymetrix^®^GeneChip^®^ Command Console^®^Software (Affymetrix Inc, Santa Clara, CA). The signal intensity of individual miRNA probes was expressed as a ratio of the internal control. Microarray results are deposited in the NCBI’s Gene Expression Omnibus.

### miRNAs for quantitative real-time PCR validation

miRNAs were selected for further validation and analysis following the criteria of significant differential expression and having predictive target genes in public databases. Five RSV infection and five healthy samples were prepared for selected miRNAs validation by quantitative real-time PCR (qRT-PCR).

Total RNA was purified using mirVana™ miRNA Isolation Kit (AM1561, Ambion, Austin, TX) and 100 ng per reaction was used for qRT-PCR analysis using the Power SYBR^®^R Green PCR Master Mix and the 7900 HT Fast RealTime PCR system (Applied Biosystems, CA). All primers (miRNA RT primer & PCR primers) were synthesized by Invitrogen (Thermo Fisher Scientific Corp., CA). Transcript expression was normalized using mammalian U6 as the endogenous housekeeping gene. QRT-PCR reaction was performed with the following conditions: 95 °C for 15 min, followed by 40 cycles at 95 °C for 15 s, and 60 °C for 60 s, and 60 °C to 90 °C for temperature ramp 2 %. Samples were analyzed in triplicates, using ABI Prism SDS2.4 software (Thermo Fisher Scientific Corporation, CA). Samples were normalized and calibrated using the 2^−ΔΔ(Ct)^ method [19].

### Bioinformatics analysis

An extensive analysis of miRNAs target genes was performed by using ten different algorithms DIANAmT, miRanda, miRDB, miRWalk, RNAhybrid, PICTAR4, PICTAR5, PITA, RNA22 and Targetscan (Table 1). The names of miRNAs were put in these databases, if there were predictive target genes of the miRNA in the database, we marked one score, if they were not predictive, we marked zero. The total score was calculated in the end. The higher the score, the more dependable the target gene was. Higher score target genes were selected for performing enriched pathways and functional analysis by the Kyoto encyclopedia of genes and genomes (KEGG) database [20] and Gene ontology miR (GOmir) [21].

### Ethics statement

For de-identification, the blood samples were codified as RSVn (for RSV infection) and CTLn (for healthy controls) to protect the privacy of individuals during all molecular studies. All patients gave informed written consent and the samples were processed under approval from the Zhongnan Hospital of Wuhan University Ethics Committee.

### Statistics

Microarray results were analyzed by the R computing environment. The Affymetrix^®^ GeneChip^®^ Command Console^®^ Software with quintiles normalization was used for pre-processing. Differential expression analysis of microarray data was assessed using fold-change method [22]. Differential expression miRNA was set by fold-change ≥2 for upper regulation and by fold-change ≤ 0.5 for downregulation [23]. Cluster data of microarray results were analyzed by hierarchical clustering with pairwise average-linkage approach. PCR results were analyzed using IBM SPSS 20.0 software (Chicago, IL). Adjusted p-values < 0.05 were considered statistically significant.
